# Chromium(VI) Removal from Aqueous Solution by Magnetite Coated by a Polymeric Ionic Liquid-Based Adsorbent

**DOI:** 10.3390/ma10050502

**Published:** 2017-05-06

**Authors:** Thania Alexandra Ferreira, Jose Antonio Rodriguez, María Elena Paez-Hernandez, Alfredo Guevara-Lara, Enrique Barrado, Prisciliano Hernandez

**Affiliations:** 1 Area Academica de Quimica, Universidad Autonoma del Estado de Hidalgo, Carr. Pachuca-Tulancingo Km. 4.5, Mineral de la Reforma, Hidalgo 42184, Mexico; alexandrafg21@gmail.com (T.A.F.); josear@uaeh.edu.mx (J.A.R.); paezh@uaeh.edu.mx (M.E.P.-H.); guevaraa@uaeh.edu.mx (A.G.-L.); 2 Departamento de Química Analítica, Facultad de Ciencias, Universidad de Valladolid, Paseo de Belén 7, Valladolid 47011, Spain; ebarrado@qa.uva.es; 3 Área de Energías, Universidad Politécnica de Francisco I. Madero, Domicilio Conocido, Tepatepec, Hidalgo C.P. 42640, Mexico

**Keywords:** chromium(VI), magnetic particles, ionic liquid, adsorption capacity

## Abstract

An evaluation of the chromium(VI) adsorption capacity of four magnetite sorbents coated with a polymer phase containing polymethacrylic acid or polyallyl-3-methylimidazolium is presented. Factors that influence the chromium(VI) removal such as solution pH and contact time were investigated in batch experiments and in stirred tank reactor mode. Affinity and rate constants increased with the molar ratio of the imidazolium. The highest adsorption was obtained at pH 2.0 due to the contribution of electrostatic interactions.

## 1. Introduction

Chromium(VI) is a highly toxic species; it is considered on the priority list of highly toxic pollutants by the Environmental Protection Agency of the United States (EPA), which has established 50 μg/L as the maximum permitted level for chromium(VI) [1].

The main source of chromium(VI) is associated with anthropogenic activities such as electroplating, textile industries, and pigments. Depending on the pH conditions and concentration of the media, this element can be found as CrO_4_^2−^, HCrO^4−^, or Cr_2_O_7_^2−^; these species are hard oxidants, and have high solubility in water, making them a potential danger to living organisms. Chromium(VI) has negative consequences for human health. Besides causing skin irritation, chromium(VI) compounds are considered carcinogenic and mutagenic from group A according to the international agency for research on cancer [2,3].

There is a wide range of techniques for the selective removal of chromium(VI) from water, such as ultrafiltration [3], liquid–liquid extraction [4], ion exchange [5], electrochemical removal [6], and in recent years, detoxification by the presence of microorganisms [7]. Nevertheless, the most widely-used technique is adsorption because of its advantages above the other techniques: high efficiency, low cost, minimum use of organic solvents, simplicity, and reusability. Chromium(VI) adsorption has been carried out with different sorbents, including clays [8], chitosan [9], nanocomposites [10], activated carbon [11], biosorbents [12,13,14,15], and recently, magnetic particles [16]. Magnetic materials have been considered useful because they can be modified to improve selectivity and adsorption processes [16], and they can also be easily separated from the media by applying an external magnetic field, minimizing secondary pollution [17,18].

Sorbents based on iron oxide particles have been used for this purpose in the past few years. In all cases, the magnetic particles’ surfaces have been modified with functional polymers in order to avoid air oxidation and the formation of aggregates in solution, also conferring selectivity and stability to the magnetic particles [19]. There are examples of the recovery of heavy metals, including Cd(II), Cu(II), Ni(II), and chromium(VI) by maghemite coated with polyethylene glycol [20], magnetic gelatins [18], catecholamine-coated maghemite nanoparticles [21], and polypyrrole-coated magnetite [19].

In addition, the presence of functional groups such as –OH and –COOH on the surface can enhance the interaction with anions due to electrostatic interactions. Treatment performed at low pH values promotes the formation of positive charges on the solid surface and favors the electrostatic attraction with negatively-charged chromium(VI) species [18].

On the other hand, the use of ionic liquids (IL) in solid phase extraction has gained interest [4]. In recent years, these compounds have been physically or chemically immobilized in solids [22]. Nano-silica has been modified with 1-butyl-3-methylimidazolium hexafluorophosphate for Pb(II) adsorption; the synthesis of the adsorbent was based on the physical adsorption of the IL on the surface of activated nano-silica by suspending the silica particles in a solution containing the IL [23]. Interaction between the sorbent and the analyte is attributed to physical interactions (Van der Waals forces, hydrogen bonding), chemical interactions (bond formation), electrostatic interactions, the formation of coordination complexes via the donor atoms, or ionic exchange [23,24]. Alternatively, IL can be immobilized using them as monomers for the preparation of polymers [25]. It has been proved that the use of IL for the adsorption of chromium(VI) enhances the desired behavior of the sorbent, improving its adsorption capacity and selectivity towards the ion of interest [26].

**Figure 1:**
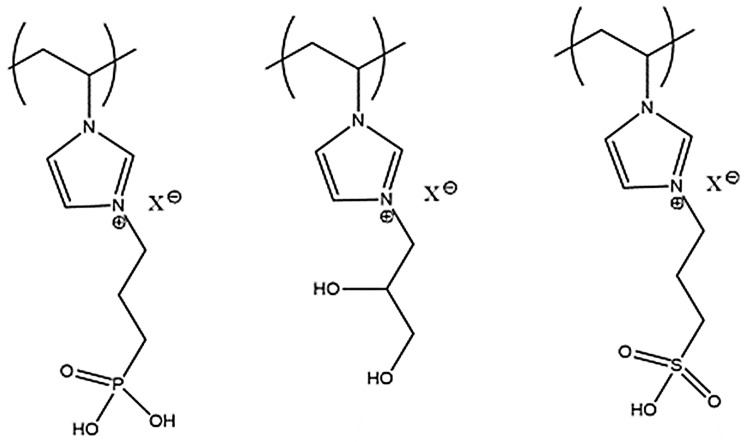
Chemical structures recently reported for cationic poly (ionic liquids) (PILs) [28].

Poly(ionic liquids) (PILs) have gained considerable attention in the past few years because these materials possess physical and chemical properties covering a wide range of applications. They are considered as multifunctional polyelectrolytes that can be used as solid ion conductors, as sorbents, and in catalysis. Yuan et al. described the synthesis of PIL-based core–shell nanoparticles using inorganic and organic cores for their use in separation techniques [27], combining the unique IL properties and the small dimension of nanoparticles that amplifies the surface features, giving rise to a new class of polymeric materials. PILs are obtained via radical polymerization of the IL monomer; some examples of PIL structures are pointed out in Figure 1 [28].

Therefore, this work proposes the synthesis of magnetic sorbents coated with polymers based on 1-allyl-3-methylimidazolium for the removal of chromium(VI) from water.

## 2. Results and Discussion

### 2.1. Structural Characterization

**Figure 2:**
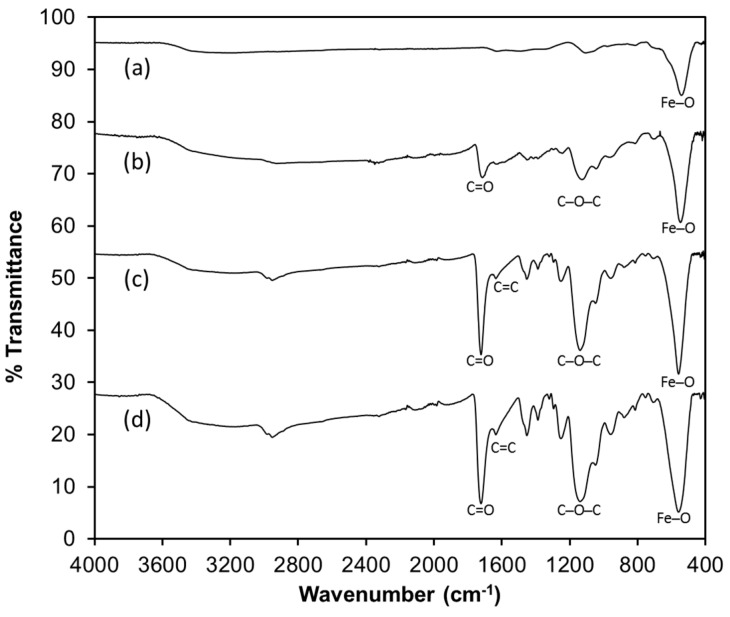
Fourier transform infrared (FTIR) spectra of the sorbents. (**a**) Fe_3_O_4_; (**b**) Fe_3_O_4_-MAA; (**c**) Fe_3_O_4_-MAA-IL; (**d**) Fe_3_O_4_-IL. IL: ionic liquid; MAA: methacrylic acid.

The synthesized sorbents were characterized by Fourier transform infrared spectroscopy (FTIR) in order to evaluate the functional groups present in the solids (Figure 2). For the magnetite (Figure 2a), a band at 560 cm^−1^ is characteristic for the bending vibration of the Fe–O bonds; this is also observed in the modified sorbents (b–d). Bands observed at 1137 cm^−1^ and 1722 cm^−1^ correspond to the presence of C–O–C and C=O groups in the magnetite-polymer (Figure 2b–d) due to the presence of ethylene glycol dimethacrylate (EGDMA) as a cross-linking agent. For the spectra of the magnetite coated with 1-allyl-3-methylimidazolium chloride as monomer (Figure 2c,d), a band at 1635 cm^−1^ characteristic of the C=C bond of the imidazolium ring is observed [25].

**Figure 3:**
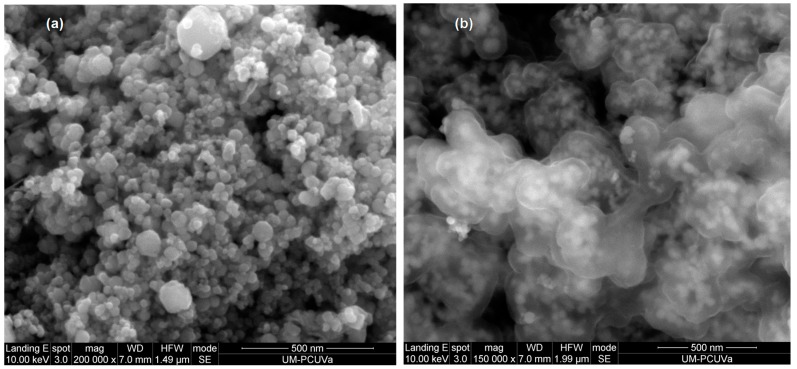
SEM images obtained of the synthesized adsorbents. (**a**) Fe_3_O_4_; (**b**) coated Fe_3_O_4_.

The morphology of the particles was studied by scanning electron microscopy. The micrograph of bare magnetite particles (Figure 3a) shows the formation of spherical particles with diameter around 50 nm. For coated magnetite particles (Figure 3b), it is possible to observe the formation of aggregates. Modifying the magnetite surface with polymer coatings gives the particles greater stability in solution and avoids air oxidation [18,19].

### 2.2. Adsorption Experiments

#### 2.2.1. Batch Studies and Effect of the Solution pH

**Figure 4:**
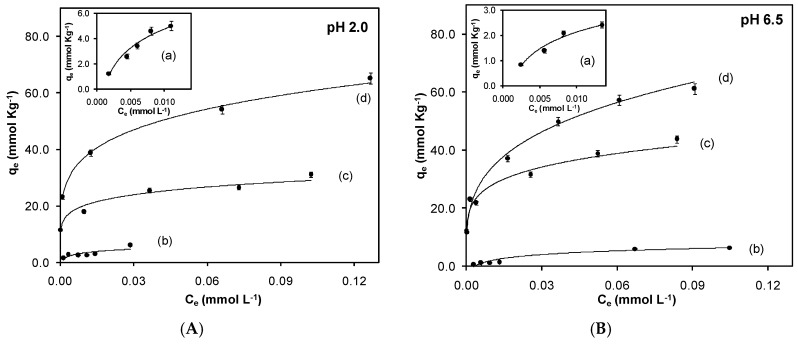
Effect of pH (**A**) 2.0 and (**B**) 6.5 on the adsorption. (**a**) Fe_3_O_4_; (**b**) Fe_3_O_4_-MAA; (**c**) Fe_3_O_4_-MAA-IL; (**d**) Fe_3_O_4_-IL.

The experiments to evaluate the equilibrium of adsorption were performed at pH values of 2.0 and 6.5 in order to evaluate the effect between the surface charge and the chromium(VI). Figure 4 shows the adsorption isotherms for the synthesized sorbents.

The adsorption isotherms for Cr(VI) show a strong dependence on the pH value, and it decreases as the pH increases as a consequence of the charge repulsion between the surface of the solid negatively-charged and the anionic species chromium(VI) CrO_4_^2−^. Adsorption exhibited a dependence on the electrostatic interactions.

It was observed that the synthesized solids Fe_3_O_4_, Fe_3_O_4_-MAA (methacrylic acid), Fe_3_O_4_-MAA-IL, and Fe_3_O_4_-IL present a significant difference in their adsorption capacity (Figure 4). For magnetite, the surface charge is neutral at pH (6.0–7.3); below this value, the surface of the magnetite is positively charged, and the predominant chromium(VI) species is HCrO_4_^−^, favoring the electrostatic attraction and also the adsorption; instead, at pH values higher than pH_pzc_, the magnetite surface acquires negative charge, causing electrostatic repulsions with the predominant chromium(VI) species CrO_4_^2−^. In the case of magnetite covered with polymer phase, the groups such as –OH and –COOH can be protonated at low pH values, causing the formation of positive charges on the surface, improving the interaction with chromium(VI) anions because of the presence of electrostatic attraction [18]. When the polymer phase is composed of the imidazolium salt, an increase in the adsorption capacity is observed. It has been reported that IL-based materials show an increase in selectivity and adsorption capacity due to anion exchange interactions [25], in this case, between the Cl^−^ of the imidazolium salt and the chromium(VI) species HCrO_4_^−^.

On the other hand, chromium(VI) can be reduced to Cr(III) in acidic solution in the presence of organic matter [29]. Complexation phenomena between carbonyl groups (C=O) and Cr(III) can also occur, as oxygen in this group is considered a strong Lewis base capable of complexation with metal cations. Then, a speciation chromium oxidation state on the solid must also be considered in order to propose the adsorption mechanism [30].

Magnetite shows a lower adsorption capacity of chromium(VI) (5.01 mmol/kg at pH 2.0) compared to the use of coated magnetic particles, with acrylic polymer (Fe_3_O_4_-MAA) showing a slight increase in the adsorption capacity (6.11 mmol/kg at pH 2.0). On the other hand, adding the imidazolium salt as functional monomer improves the capacity of the solid to retain the chromium(VI) anions, as shown in the isotherms for Fe_3_O_4_-MAA-IL and Fe_3_O_4_-IL. The maximum adsorption capacity is 65.16 mmol/kg for Fe_3_O_4_-IL carrying out the adsorption process at pH 2.0.

Once the isotherms were obtained, Scatchard plots were used to calculate the values of affinity constants for each solid. The values obtained for affinity constants at pH 2.0 for Fe_3_O_4_, Fe_3_O_4_-MAA, Fe_3_O_4_-MAA-IL, and Fe_3_O_4_-IL were 40.7, 8.13, 5.01, and 1.41 μM, respectively. An improvement in the affinity of the solid towards chromium(VI) was observed by increasing the molar ratio of the imidazolium salt in the polymer phase. The solid with a molar ratio of 4.3:2.0:1.0 (Fe_3_O_4_:EGDMA:IL) was the one that presented greater adsorption capacity and the highest affinity at pH value of 2.0. Based on the results obtained, pH 2.0 was chosen to carry out kinetic studies for the modified sorbents.

#### 2.2.2. Adsorption Kinetics: Stirred Tank Experiments

**Figure 5:**
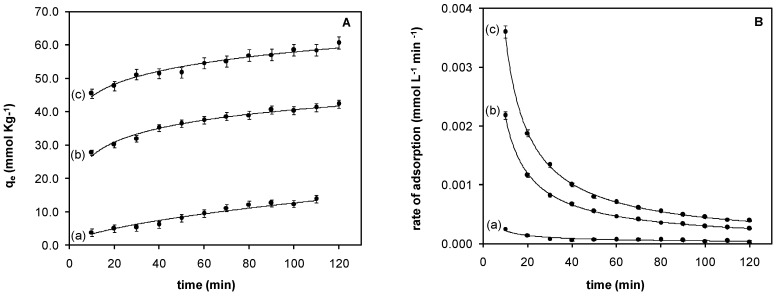
Adsorption kinetics: (**A**) Adsorption capacity with respect to contact time and (**B**) Rate of adsorption with respect to contact time (pH 2.0); (**a**) Fe_3_O_4_-MAA; (**b**) Fe_3_O_4_-MAA-IL; (**c**) Fe_3_O_4_-IL.

The chromium(VI) adsorption with respect to contact time was evaluated at pH 2.0. The results are presented in Figure 5A. The adsorption of chromium(VI) increases with contact time, achieving values of at least 70% in the first 120 min with the solids containing IL in the polymer phase. Removal efficiency decreases as follows: Fe_3_O_4_-IL > Fe_3_O_4_-MAA-IL > Fe_3_O_4_-MAA. The highest chromium(VI) uptake was 90.94% with respect to the initial Cr(VI) concentration employed.

Adsorption kinetics was evaluated using pseudo-first-order kinetic model, and results have a good linear correlation. The value of the rate constant (*k*) was calculated from the slope of the linear plot of ln(*q_e_ − q_t_*) versus time (*t*), as shown in Equation (5). Adsorption rate constants and correlation coefficient for each solid are given in Table 1. In all cases, results had a good linear correlation adjusting to a pseudo-first-order process. According to the results presented in Figure 5B and in Table 1, the adsorption rate increases with the IL content and decreases over time due to the saturation of sites available for interaction or ion exchange. Rate constants of other chromium(VI) sorbents reported are summarized in Table 1. The synthesized solids in this work have higher rate constants.

According to the results presented in Figure 5b and in Table 1, the adsorption rate increased with the IL content, and decreased over time due to the saturation of sites available for interaction or ion exchange.

Rate constants of other chromium(VI) sorbents reported are summarized in Table 1. These studies indicate that chromium(VI) adsorption obeys a pseudo-first-order kinetic model; however, the synthesized solids in this work have higher rate constants.

## 3. Materials and Methods

### 3.1. Materials

All solutions were prepared with deionized water (Millipore system) with a resistance of 18.2 MΩ cm or greater. All chemicals used were reagent grade. Potassium dichromate (K_2_Cr_2_O_7_) was purchased from Sigma Aldrich (St. Louis, MO, USA), and a stock solution of 500 mg/L of chromium(VI) was prepared. Chromium(VI) solutions were prepared from dilutions from the stock solution. 1,5-Diphenylcarbazide, sodium persulfate (Na_2_S_2_O_8_), ethylene glycol dimethacrylate (EGDMA), methacrylic acid (MAA), 1-allyl-3-methylimidazolium chloride (IL), iron (II) sulfate heptahydrate (FeSO_4_∙7H_2_O), sodium hydroxide, sulfuric acid, and methanol were also purchased from Sigma Aldrich.

### 3.2. Synthesis and Characterization of Polymer-Coated Fe_3_O_4_ Particles

Precipitation method was employed for the preparation of Fe_3_O_4_ particles; 12.96 mmol (3.6 g) of FeSO_4_∙7H_2_O were dissolved in 100 mL of deionized water, and NaOH (6 M) was added until pH 10.0 ± 0.2 and dark green color were obtained. The suspension was stirred at 300 rpm, aerated, and heated at 100 °C during 45 min, keeping pH value at 10.0 ± 0.2. Magnetic particles were obtained according to the reaction represented in Equation (1) [20].

(1) $${\mathrm{Fe}}^{2+}+2{\;\mathrm{OH}}^{-}\to \mathrm{Fe}{\left(\mathrm{OH}\right)}_{2}↓\;3\;\mathrm{Fe}{\left(\mathrm{OH}\right)}_{2}\;+0.5{\;O}_{2}\to \mathrm{Fe}{\left(\mathrm{OH}\right)}_{2}\;+2\;\mathrm{FeOOH}\;+{\;H}_{2}O\;\mathrm{Fe}{\left(\mathrm{OH}\right)}_{2}\;+2\;\mathrm{FeOOH}\;\to {\mathrm{Fe}}_{3}O_{4}\;+2{\;H}_{2}O$$

The resulting suspension with a black precipitate was separated using a magnet to retain the magnetic particles, and the supernatant was decanted. Magnetite was washed with deionized water (3 × 10 mL) followed by cold ethanol (2 × 10 mL). Magnetite was dispersed in methanol (15 mL), and it was transferred into a ball flask containing methacrylic acid (MAA), IL monomer, and EGDMA. Fe_3_O_4_ (4.3 mmol) and EGDMA (4 mmol) were kept constant while varying the concentration of MAA (0–2 mmol) and IL (0–2 mmol). The mixture was stirred for 15 min. Then, 0.5 mmol of solid Na_2_S_2_O_8_ (0.12 g) was added as radical initiator, and a reflux system was mounted. The temperature was ramped from room temperature to 60 °C over the first 2 h, and maintained for 2 h [31]. The obtained solid was washed with deionized water, and left in the oven at 60 °C for 8 h to dry. The dried particles were kept in a desiccator prior to use. The resulting sorbents are composed as follows, considering the molar ratio mentioned above. Fe_3_O_4_, Fe_3_O_4_-MAA, Fe_3_O_4_-MAA-IL, Fe_3_O_4_-IL (Table 2).

Once the sorbents were synthesized, they were characterized by Fourier transform infrared spectroscopy (FT-IR) in a Perkin-Elmer Frontier spectrometer (Waltham, MA, USA) between 4000 and 400 cm^−1^ in order to identify the functional groups in the structure. Micrographs of the sorbents were taken using scanning electron microscopy (FEI Model Quanta 200 F, Amsterdam, The Netherlands).

### 3.3. Adsorption Experiments

#### 3.3.1. Batch Studies

Batch studies were performed by mixing the synthesized sorbents (8.0 mg) with 10 mL of chromium(VI) solutions (0–20 mg/L). The contact time was 30 min in a multi-wrist shaker (model 3589). Different factors, such as solution pH and contact time were evaluated. Chromium(VI) adsorption was first studied at two pH values (2.0 and 6.5) to investigate the dependence on solution pH. Sulfuric acid 0.01 M and sodium hydroxide 0.01 M were used for pH adjustment.

Once the contact time was completed, the magnetic sorbent was recovered by an external magnet, and the supernatant was decanted. Adsorption capacity values were calculated from change in the concentration of the chromium(VI) in the solutions employed using the diphenylcarbazide method measuring at 540 nm in a HACH spectrophotometer (DR-2700, Dusseldorf, Germany). To describe the equilibrium of adsorption, the data was fitted to an adsorption isotherm by plotting the remaining concentration of chromium(VI) with respect to the adsorbed chromium(VI), which is calculated according to Equation (2):

(2) $$q_{e}=\frac{\left(C_{0}-C_{e}\right)V}{w}$$

where *q_e_* is the adsorbed chromium (mmol/kg), *C*_0_ and *C_e_* are initial and final concentrations, respectively (mmol/L), *V* is the volume of the solution (L), and *w* is the sorbent mass (kg).

Affinity constant values were calculated using the Scatchard method by plotting *q_e_*/*C_e_* versus *C_e_* (where *q_e_* is expressed in terms of mol/kg and *C_e_* in terms of mol/L) [32].

#### 3.3.2. Semi-Continuous System

Adsorption kinetic studies were carried out in a semi-continuous system implemented to calculate the saturation rate of the synthesized sorbents. One-hundred milliliters of 2.0 mg/L chromium solution were mixed with the different sorbents individually (80.0 ± 0.3 mg). Volumes of 2.0 mL were taken every 10 min for chromium(VI) measurement. The experiments were performed in a stirred tank mode using a stir-pak laboratory stirrer from Cole-Parmer with a helix stirrer from multi-craft.

The velocity for a first-order kinetic model for the adsorption obeys Equation (3) [33]:

(3) $$\frac{dC_{e}}{dt}=kC_{e}$$

Lagergren proposed an adaptation of the equation starting from the concentration of adsorbed chromium(VI); Equation (4) is the velocity equation for a pseudo-first-order reaction (Equation (4)), where the velocity of the adsorption process depends on the velocity constant (*k*), the maximum adsorbed concentration of chromium(VI) (*q_e_*), and the adsorption at time *t* (*q_t_*) with the units described above.

(4) $$\frac{dq_{t}}{dt}=k\left[q_{e}-q_{t}\right]$$

Equation (4) was integrated with respect to the initial and final conditions, and Equation (5) was obtained where *t* is the time when the sample was taken.

(5) $$\ln\left(q_{e}-q_{t}\right)=\ln q_{e}-kt$$

By plotting ln(*q_e_* − *q_t_*) versus t from the pseudo-first-order equations for each solid, it is possible to calculate the velocity constant (*k*) for the adsorption and obtain the velocity equation.

## 4. Conclusions

Magnetic sorbents with potential use for chromium(VI) removal were synthesized and evaluated. Adsorption exhibited a clear dependence on the pH of the chromium solution. Highest adsorption capacity was obtained in acidic solutions (pH 2.0), and a speciation of chromium oxidation state is required to identify the adsorption mechanism. Fe_3_O_4_-IL was the solid that had the highest affinity and the best adsorption capacity. The rate constants for the adsorption process fit to a pseudo-first-order equation, and the value of the constant increased by increasing the IL molar ratio. The use of the ionic liquid-modified magnetic particles for chromium(VI) removal is feasible, economically attractive, and environmentally-friendly by diminishing secondary pollution because of their easy separation from the medium.

## Figures and Tables

**Table 1:** Kinetic data obtained from stirred tank experiments at pH 2.0.

Sorbent	Rate Constant min^−1^ (×10^−3^)	R^2^	Reference
Fe_3_O_4_	6.56 ± 0.75	0.98	-
Fe_3_O_4_-MAA	25.40 ± 5.50	0.93	This work
Fe_3_O_4_-MAA-IL	25.30 ± 3.20	0.97	-
Fe_3_O_4_-IL	27.80 ± 6.10	0.94	-
Activated carbon derived from acrylonitrile–divinylbenzene copolymer	5.99	0.8369	[11]
*Acinetobacter junii* biomass	18.00	0.991	[12]

**Table 2:** Molar ratio for the synthesized sorbents (mmol); EGDMA: ethylene glycol dimethacrylate.

Sorbent	Fe_3_O_4_	EGDMA	MAA	IL
Fe_3_O_4_	4.3	-	-	-
Fe_3_O_4_-MAA	4.3	4.0	-	-
Fe_3_O_4_-MAA-IL	4.3	4.0	2.0	0.0
Fe_3_O_4_-IL	4.3	4.0	0.0	2.0
